# MSFC: a new feature construction method for accurate diagnosis of mass spectrometry data

**DOI:** 10.1038/s41598-023-42395-5

**Published:** 2023-09-21

**Authors:** Xin Feng, Zheyuan Dong, Yingrui Li, Qian Cheng, Yongxian Xin, Qiaolin Lu, Ruihao Xin

**Affiliations:** 1 School of Science, Jilin Institute of Chemical Technology, Jilin, 130000 People’s Republic of China; 2 State Key Laboratory of Inorganic Synthesis and Preparative Chemistry, College of Chemistry, Jilin University, Changchun, 130012 People’s Republic of China; 3 College of Information and Control Engineering, Jilin Institute of Chemical Technology, Jilin, 130000 People’s Republic of China; 4 College of Computer Science and Technology, and Key Laboratory of Symbolic Computation and Knowledge Engineering of Ministry of Education, Jilin University, Changchun, 130012 People’s Republic of China; 5 College of Business and Economics, Australian National University, Canberra, ACT 2601 Australia; 6 School of Artificial Intelligence, Jilin University, Changchun, 130012 People’s Republic of China

**Keywords:** Data processing, Computational biology and bioinformatics, Machine learning

## Abstract

Mass spectrometry technology can realize dynamic detection of many complex matrix samples in a simple, rapid, compassionate, precise, and high-throughput manner and has become an indispensable tool in accurate diagnosis. The mass spectrometry data analysis is mainly to analyze all metabolites in the organism quantitatively and to find the relative relationship between metabolites and physiological and pathological changes. A feature construction of mass spectrometry data (MSFS) method is proposed to construct the features of the original mass spectrometry data, so as to reduce the noise in the mass spectrometry data, reduce the redundancy of the original data and improve the information content of the data. Chi-square test is used to select the optimal non-redundant feature subset from high-dimensional features. And the optimal feature subset is visually analyzed and corresponds to the original mass spectrum interval. Training in 10 kinds of supervised learning models, and evaluating the classification effect of the models through various evaluation indexes. Taking two public mass spectrometry datasets as examples, the feasibility of the method proposed in this paper is verified. In the coronary heart disease dataset, during the identification process of mixed batch samples, the classification accuracy on the test set reached 1.000; During the recognition process, the classification accuracy on the test set advanced to 0.979. On the colorectal liver metastases data set, the classification accuracy on the test set reached 1.000. This paper attempts to use a new raw mass spectrometry data preprocessing method to realize the alignment operation of the raw mass spectrometry data, which significantly improves the classification accuracy and provides another new idea for mass spectrometry data analysis. Compared with MetaboAnalyst software and existing experimental results, the method proposed in this paper has obtained better classification results.

## Introduction

Metabolomics plays a crucial role in biological systems and aims to investigate the development of biology and disease by studying all metabolites in biological samples^1–3^. Compared with upstream genomics, transcriptomics, and proteomics research, metabolomics can expand from the field of genes to the area of small molecules, realize the integration of small molecules into bioinformatics, and combine other omics technologies to discover systematic biomedicine research in the field. Nowadays, as one of the most widely used platforms of metabolites, mass spectrometry can repeatedly detect thousands of metabolites from cells^4–6^, tissues, and biological fluids, assist biomedical research in detecting multiple feature subsets, and is of great significance in promoting biomarker screening^7–9^, pathological research^10–12^, and drug development^13–15^. In metabolomics, researchers used mass spectrometry to analyze changes in the levels of metabolites in organisms to study the dynamic development of organism physiology. Although histopathology is still the gold standard for modern disease diagnosis, proteomics and metabolomics studies based on mass spectrometry technology have been widely used in the diagnosis of various diseases, which can Achieve early and accurate diagnosis of clinical-level diseases^16^. Exploring the metabolite molecules corresponding to biomarkers in the metabolic process of conditions will help elucidate the mechanism of disease in the disease process and find marker molecules that play an essential role in the occurrence and development of diseases^17^.

Due to the difference in sample sampling time, the sample length is different. At the same time, mass spectrometry data has a high feature dimension, which contains much biological information and a large amount of redundant information and noise. How to remove noise and errors from such high-dimensional features and extract useful biological information has become one of the leading research points of bioinformatics^18^. Therefore, there is an urgent need to find an efficient data processing method to extract and analyze large-scale multidimensional raw spectral data, especially raw Mass spectral data generated from clinical samples.

At present, many machine learning (ML) models have been widely used for accurate diagnosis of diseases and biomarker mining under mass spectrometry data, such as support vector machine (SVM), random forest (RF), Bayesian neural network (NB), and Linear discriminant analysis (LDA) has achieved good results^19–22^. These machine learning methods are suitable for preprocessed mass spectrometry data, but differences in preprocessing pose a significant challenge for any comparative analysis. As a critical step in sample classification, data preprocessing is used to align mass spectrometry sample data and eliminate noise errors and redundant information from original mass spectrometry data. However, taking inappropriate preprocessing operations will result in the loss of useful information in the data. Therefore, proper preprocessing methods should be used when performing data preprocessing^23^.

This paper mainly aims at the uneven raw data containing noise and uses two-dimensional mass spectrometry data to achieve accurate diagnosis of diseases and mining of biomarkers. The features of mass spectrometry data are constructed by the data preprocessing method, and the feature selection is carried out by the chi-square test method. In training ten supervised learning models, the model's classification effect is evaluated through various evaluation indicators.

## Materials and methods

### Experimental design

**Figure 1:**
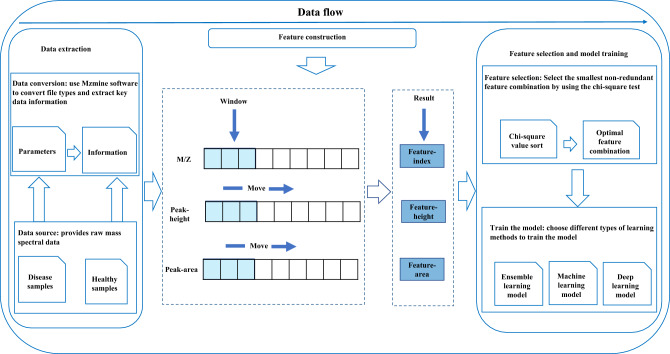
Experimental flow chart.

A step-by-step overview of the data processing in this paper is shown in Fig. 1. The experiment is mainly divided into three main steps. The first step uses MZmine to extract the critical information in the original RAW file. In the second step, the sliding window method is used to construct the features of the original mass spectrum data, and the M/Z dimension is used as the window index to realize the feature construction of the peak area and peak height, so that each sample has the same length in the M/Z dimension, and at the same time, the noise and redundant information in the data are removed. The third step is to filter out the minimum non-redundant feature subset by chi-square test and evaluate the classification effect through various evaluation indicators on ten supervised learning models.

### Summary of the dataset

The datasets used in this experiment mainly include the colorectal and liver datasets from Proteome Xchange and the coronary heart disease datasets provided by Thermo Fisher Scientific. Table 1 presents an introduction to the coronary heart disease and rectal liver sample datasets.

**Table 1:** Dataset introduction.

Disease	Dataset Ids	Raw file	Sample
			$$($$positive, negative$$)$$
Coronary heart disease	CHD_serum_102samples	102	21 $$($$1-batch$$)$$
			20 $$($$1-batch$$)$$
			38 $$($$2-batch$$)$$
			23 $$($$2-batch$$)$$
Colorectal liver metastasis tissue	PXD008383	60	30
			30

The first dataset was provided by Thermo Fisher Scientific in Massachusetts and included plasma and urine samples from 59 coronary heart disease (CHD) patients and 43 healthy controls (Control)^24^. The plasma data is divided into two batches, in which 1-Batch contains 21P samples and 20N samples, and 2-Batch contains 38P samples and 23N samples. The pathogenesis of coronary heart disease is studied by identifying new biomarkers in plasma (Dataset connection: https://datadryad.org/stash/dataset/doi:10.5061%2Fdryad.s8k81).

The second dataset is from the Proteome Xchange platform, including 30 colorectal liver metastasis (CRLM) samples from the same patient with colorectal liver metastasis and 30 adjacent normal tissue samples (Control)^25^. The liver metastases of the same colorectal liver metastases and their adjacent tissue samples have certain similarities in the related proteins. Still, they have significant differences in the number of distributions, and the liver metastases are much more extensive in some essential proteins. Therefore, try to use the critical information of its high-precision mass spectrometry samples to distinguish the two samples through machine learning algorithms. (Dataset connection: http://proteomecentral.proteomexchange.org/cgi/GetDataset?ID=PXD008383).

### Feature construction

**Figure 2:**
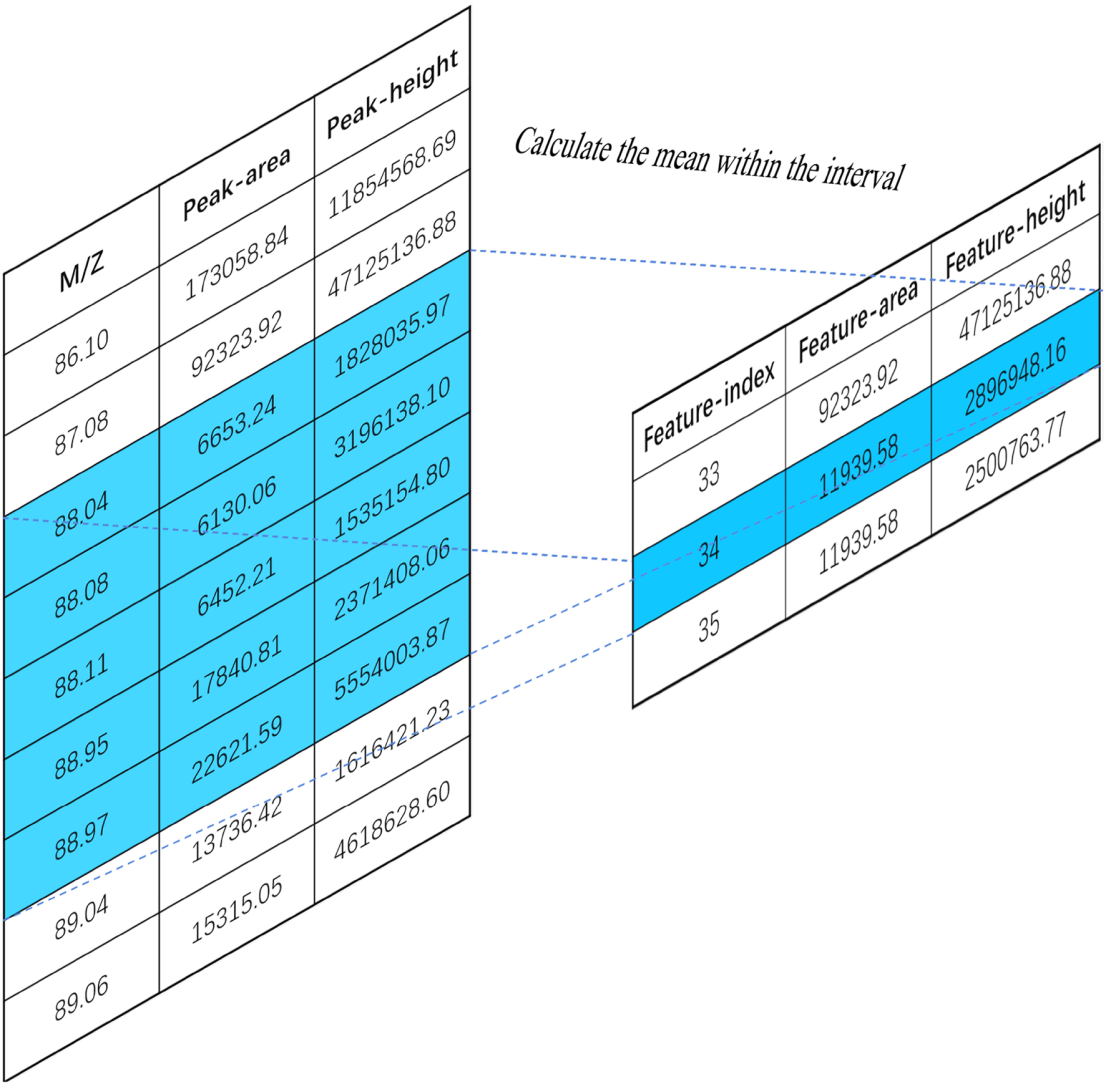
Schematic diagram of MSFS method.

In the process of mass spectrometry data collection, the collection length of each sample is inconsistent, and the collected data is relatively sparse, so it is necessary to find a way to align the mass spectrometry data. In addition, there is external interference in the process of mass spectrometry data acquisition, and the collected data will contain a lot of noise. This feature construction method can align samples with different lengths, reduce the noise in mass spectrometry data and reduce the redundancy of original data. Figure 2 is a schematic diagram of MSFS feature construction principle. MSFS has realized the characteristic structure of peak height and peak area in the mass-to-core ratio (M/Z) range. The basic idea is to scan the original mass spectrum and compare the phases of the data. Neighbors are grouped into data bins to reduce the dimension of data. Then select the representative members of each group, representing the mass-to-charge ratio, peak height and peak area of the whole group. Using M/Z as the window index, the average value of peak height and area in the window is calculated. The constructed M/Z value is taken as the characteristic index, and the average value of peak height and peak area in the window is taken as the characteristic value. The difficulty in the construction of sliding window features lies in choosing a suitable width of sliding window to scan mass spectrometry data. If the window is too large, the feature dimension will not be significantly reduced, and the redundant information in the original data can not be removed well. If the window is too small, the feature dimension reduction is not obvious, and the redundant information in the original data can not be removed well. Suppose the window width is set too large. In this case, a lot of different information in the window will be eliminated, so the reduced data can not fully express all kinds of information between samples. Setting an appropriate window size can eliminate redundant information in the original data and effectively extract the model classification results.

### Feature selection method based on chi-square test

Data feature selection is widely used in extensive data mining, analysis, and machine learning. Especially in high-dimensional mass spectrometry data, selecting important data features is a critical factor in identifying disease biomarkers. The chi-square test is a conventional feature selection algorithm used for feature selection in different research works^26^.

The Chi-square test is a statistical analysis method specially used for counting data in statistical analysis. Using the ${\chi }^{2}$ distribution and degrees of freedom, the probability P of the current statistic under H0 is obtained. The Chi-square test can measure the correlation between feature F and category ${L}_{i}$. Assuming that F and ${L}_{i}$ obey the ${\chi }^{2}$ distribution with one degree of freedom, the calculation formula of the chi-square test is:

1 $${\chi }^{2}(F,{L}_{i})=\frac{{(AD-CB)}^{2}}{(A+C)(B+D)(A+B)(C+D)}$$

Among them, F is the feature; ${L}_{i}$ is a specific category; A represents the number of subsets with feature F in ${L}_{i}$; B represents the number of subsets that do not belong to the ${L}_{i}$ category and include feature F; C represents the number of subsets belonging to ${L}_{i}$ but not including feature F, D denotes the number of subsets that neither belong to ${L}_{i}$ nor have feature F. When ${\chi }^{2}(t,{ c}_{i})=0$, the features F and ${c}_{i}$ are independent, and the greater the value of χ^2^, the stronger their correlation. Get a sorted list of features according to the χ^2^ value, then select features according to the list. The chi-square test is used for feature selection, and the chi-square value estimates the importance of high-dimensional data features. This method can quickly find the relevant information in the data features and select the feature subset with the most significant correlation and the least redundancy^27^.

### Supervised learning algorithms

In order to verify the original mass spectrum, Table 2 lists the salient features screened by Chi-square test method, and classifies the results on seven traditional machine learning algorithms, two integrated learning frameworks and one deep learning algorithm. Model training. Regression analysis (LR)^28^ fits the relatively linear data with less loss and enables the fitted model to better predict the data. A Support Vector Machine (SVM)^29^ defines a linear classifier with the most significant margin in the feature space, capable of separating samples from two different classes. When predicting a new sample, K-Nearest Neighbor (KNN)^30^ judges which category the sample belongs to according to which type the K samples closest to the sample belong. The decision tree (DT)^31^ obtains the final result through the decision-making process and discrimination of the problem by the tree. Random Forest (RF)^32^ is a method that uses multiple decision trees to train, classify and predict sample data. Lightweight Gradient Boosting Machine (LightGBM)^33^ is a fast, distributed, high-performance decision tree-based gradient boosting algorithm that starts with weak models and trains models iteratively, each model adding to the predictions of previous models to produce a reliable Overall forecast. Naive Bayes (NB)^34^ estimates the conditional probability of each category by calculating the frequency of occurrence of each type in the training samples and dividing each feature attribute, and records the results, and finally outputs the classification of the samples. Bagging^35^ estimates a sample by averaging the posterior probabilities of the non-uncounted nodes it falls into. Reinforcement learning (Adaboost)^36^ trains different classifiers on the data set and then combines these weak classifiers to form a more robust final classifier. The classification effect is better than the conventional weak classifier. Classifier model effect. The deep learning model is a multilayer perceptron (MLP)^37^ or a multilayer perceptron. This forward-structured artificial neural network maps a set of input vectors to a group of output vectors and is used to fit complex functions or solve problems—classification problem.

**Table 2:** Ten supervised learning models used in this paper.

Algorithm category	Algorithm	Parameter
Conventional machine-learning algorithms	Random forest (RF)	n_estimators = 10
	Decision tree (DT)	Penality = 1.0
	Support vector machine (SVM)	cache_size = 200
	K-nearest neighbors (KNN)	n_neighbors = 5
	Logistic regression (LR)	Penalty = l2
	Light gradient boosting machine (LightBGM)	n_estimators = 100
	Naive Bayes (NB)	Alpha = 1.0
Ensemble-learning frameworks	Bagging (Bagging)	n_estimator = 100
	Adaptive boosting (AdaBoost)	n_estimators = 50
Deep-learning algorithms	Multilayer perceptron (MLP)	Layer = 32

### Evaluation method of performance

To evaluate the prediction model, this paper uses seven evaluation indicators widely used in the medical field to assess the classification effect of ten models, including Precision, Specificity, Sensitivity, Matthew Coefficient (MCC), Accuracy, F1-score, and Area under roc curve (AUC)^38^.

To evaluate the feasibility of the method proposed in the paper, the precision rate, sensitivity, specificity, Matthew coefficient, and accuracy rate were evaluated, respectively. The calculation formula is as follows:

2 $$Precision=\frac{TP}{\left(TP+FP\right)}$$

3 $$Specifity=\frac{TN}{\left(TN+FP\right)}$$

4 $$Sensitivity=Recall=\frac{TP}{\left(TP+FN\right)}$$

5 $$MCC=\frac{TP*TN-FP*FN}{\sqrt{\left(TP+FP\right)*\left(TP+FN\right)*\left(TN+FP\right)\left(TN+FN\right)}}$$

6 $$Accuracy=\frac{TP+TN}{TP+TN+FP+FN}$$

Among them, true negatives (TN) are the number of predicted healthy labels that match the true healthy labels; true positives (TP) are the number of predicted disease labels that match the actual disease labels; false negatives (FN) refer to the number of patients with disease The number of sick labels that are misidentified as healthy; false positives (FP) are the number of healthy labels that are predicted to be diseased.

F1-score is the harmonic mean of precision rate and recall rate. The F1 score is calculated to measure the classification performance of samples of different categories. The calculation formula is:

7 $$F1 score=2*\frac{Precision*Recall}{\left(Precision+Recall\right)}$$

AUC refers to the area covered by the ROC (receiver operating character) curve. Since it is difficult for ROC to reflect the difference between models, AUC can remember the difference between models as a numerical value. The closer the AUC is to 1, the better the performance of the model^39^. Calculated as follows:

8 $$AUC=\frac{\sum_{{x}_{i}\in positiveclass}rank{x}_{i}-\frac{M\left(M+1\right)}{2}}{M*N}$$

Among them, positive class indicates that the category is a positive example, ${x}_{i}$ is the ith sample, rank is the order of the probability of being a positive example in the model prediction instance, M is the actual number of positive examples, and N is the exact number of negative examples.

## Results and discussion

### Data preprocessing

Considering that the original mass spectrometry data set is too large and inconvenient for analysis, it is necessary to use open sources of mass spectrometry software such as MZmine2.53, Raw Converter, and MSConver for sampling to extract detailed key data information in mass spectrometry. The main steps of sampling through MZmine2.53 mass spectrometry software in this paper are: (1) Input the original Raw file into MZmine2.53 software and save important mass information through mass detection; (2) Set the mass detector to exact Mass for sampling, set the noise level to 0, and finally, generate chromatographic column information through ADAP Chromatogram builder, to ensure that all data are complete; (3) Export all mass spectrometry information related to this experiment from the generated feature lists, and the exported CSV file contains 24 columns Mass spectral information, where important information includes Mass, retention-time, peak-height, and peak-area. These raw data are sequentially transformed into regular feature matrices through a series of subsequent preprocessing methods for subsequent supervised learning models.

### Feature construction of mass spectrometry data

In the process of data collection, the length of each sample is different because of the different sampling time. In order to maintain the consistency of each sample in the M/Z dimension and construct suitable data for the machine learning model, it is necessary to use feature engineering method to construct and align the original data. Due to the difference of sampling time in the public coronary heart disease data set, the data set is divided into two batches. In this part of the experiment, the difference between the two batches was ignored and the samples of the two batches were mixed. The peak area and peak height of M/Z dimension are constructed by MSFS method, and the M/Z values in all samples are traversed, and the minimum and maximum M/Z values of 54 and 1223 are obtained. The minimum value and the maximum value are taken as the start value and the end value of the sliding window. Then, by setting the sliding window step size, the average of peak area and peak height in each sample window is taken as the feature value after feature construction.

Taking two mixed coronary heart disease data sets as the research objects, the training set and the test set are divided according to the ratio of 2:1, the step size of the sliding window is 0.1, and the feature construction is carried out on the original data. The chi-square test is used to screen the minimum non-redundant feature combination. Training is performed on ten supervised learning models, and the classification results are evaluated by various evaluation metrics (Table 3). The optimal classification results on Peak-area and Peak-height when the sliding window step size is 0.1. On Peak-area, when selecting the KNN model, when there are three features, the evaluation index ACC is 1.000. On Peak-height, when selecting the bagging model, when there are three features, the evaluation index ACC is 1.000. Through the experimental results on Peak-area and Peak-height, it can be concluded that good classification results can be obtained after using the sliding window to construct the original mass spectral data.

**Table 3:** When the sliding window is 0.1, the optimal classification results.

Peak type	Feature number	Best model	ACC
Area	3	KNN	1.000
Height	3	Bagging	1.000

The 1.20.3 version of Numpy and the 1.3.4 version of Pandas were used to process the data, using the CH2 feature screening algorithm provided in the 0.24.2 version of Sklearn and ten kinds of Supervised learning algorithms (AdaBoost, Bagging, LightGBM, MLP, LR, KNN, SVM, NB, DR, RF).

### Effect of different sliding window sizes on the results

Under different window steps, the data exhibits different distributions, which provide different degrees of information for the machine learning model. This part compares the feature information extracted from five sliding windows with different step size parameters and finally determines the optimal sliding window step size parameters through the classification results on ten supervised learning models. Table 4 shows that with the increase of the window step size parameter, the more original features contained in the M/Z dimension of each window, the original sparse feature information also increases with the increase of the window step size, the peak value, and peak area in the window become denser. As the sliding window increases, the missing values on positive and negative samples also become sparser.

**Table 4:** Statistics of sample length and missing values on positive and negative samples after feature construction under different window steps.

Window sizes	Sample length	Average missing value on health	Average missing value on disease
0.1	11,699	10,739	10,736
0.5	2330	1694	1689
1.0	1169	693	691
1.5	779	436	434
2.0	584	313	311

To explore the effect of different sliding window step sizes on sample prediction, the same data preprocessing, feature screening, and model validation methods are used throughout the experiment, under the condition that only the window step size is changed. The influence of five different window steps on the classification results was verified on the coronary heart disease data set to determine the optimal window parameters. Through Table 5, comparing the classification results of five different steps, the classification accuracy of each scale window can reach one under the different number of features. Still, the main difference is that fewer features are used to achieve the best classification effect, which also indicates that the window size setting is too large or too small to extract the difference information between samples well.

**Table 5:** Model and number of features for optimal classification results under different window step sizes.

Peak type	Window size	Feature number	Best model
Area	0.1	3	KNN
	0.5	9	Bagging
	1.0	2	AdaBoost
	1.5	6	AdaBoost
	2.0	7	LightGBM
Height	0.1	3	Bagging
	0.5	8	LR
	1.0	2	Bagging
	1.5	7	RF
	2.0	9	NB

**Figure 3:**
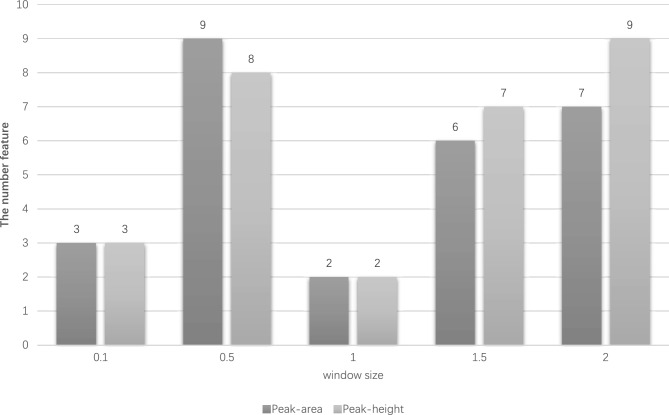
Prediction accuracy changes with window size.

As shown in Fig. 3, setting an appropriate window step size can eliminate redundant information in the original data and effectively extract the difference between coronary heart disease and normal samples. By comparing the classification results under different sliding window parameters, it is verified that when the sliding window step size is 1 (a total of 1169 windows), it is more suitable for the coronary heart disease data set. The window under this step size can reduce the noise in the original data and extract the crown Differential information between heart disease samples and healthy samples.

### Independent test validation with in the dataset

This part of the experiment uses the widely used mass spectrometry software MetaboAnalyst 5.0 version to analyze the public coronary heart disease data set. The results are compared with the method proposed in this paper^40^. Table 6 shows that, on Peak-area and Peak-height, each evaluation index of the model is 1.000 for two features, and each evaluation index is higher than the results tested on MetaboAnalyst5.0 software.

**Table 6:** Comparison results with MetaboAnalyst software.

Evaluation indicators	Method	Area	Height
Sn	MetaboAnalyst	0.983	0.983
	Self-Method	1.000	1.000
Sp	MetaboAnalyst	0.977	0.977
	Self-Method	1.000	1.000
Pre	MetaboAnalyst	0.983	0.983
	Self-Method	1.000	1.000
Acc	MetaboAnalyst	0.983	0.983
	Self-Method	1.000	1.000
MCC	MetaboAnalyst	1.000	1.000
	Self-Method	1.000	1.000
F1	MetaboAnalyst	0.983	0.983
	Self-Method	1.000	1.000
AUC	MetaboAnalyst	-	-
	Self-Method	1.000	1.000

Divide the coronary heart disease data set according to the ratio of the comparison papers (training set: healthy 29, coronary heart disease 29; test set: healthy 14, coronary heart disease 30)^41^, and verify the classification results under the optimal window step size. When there are two features on peak area and peak height, on the bagging model, the AUC is 1.000. The AUC of the comparison paper is 0.999 when there are eight features. Compared with the results of the comparison paper, the method proposed in this paper uses fewer features to achieve the same results as the comparison paper, proving the feasibility of the method proposed. The experimental results show that the method proposed in this paper has superior performance in distinguishing coronary heart disease and normal samples.

### Significant difference analysis of features on positive and negative samples

With a sliding window of 1 as the step size, using the chi-square test, Peak-area and Peak-height screen out the two feature combinations with the most considerable chi-square value on the data of two batches mixed and two batches of mutual verification, respectively. Using visualization, use these feature combinations to draw scatter plots and further verify the classification effect between coronary heart disease samples and healthy samples. At the same time, it is confirmed that batch differences between batches 1 and 2 due to differences in collection time are generated on the coronary heart disease data set. Figure 3-2 is a scatter plot drawn by the two features (F_254 and F_59, F_59 and F_256, F_59 and F_115, F_59 and F_462, F_59 and F_313) with the smallest chi-square value in the feature combination corresponding to the optimal classification results of the two batches of mixed samples and batched samples on Peak-area and Peak-height.

**Figure 4:**
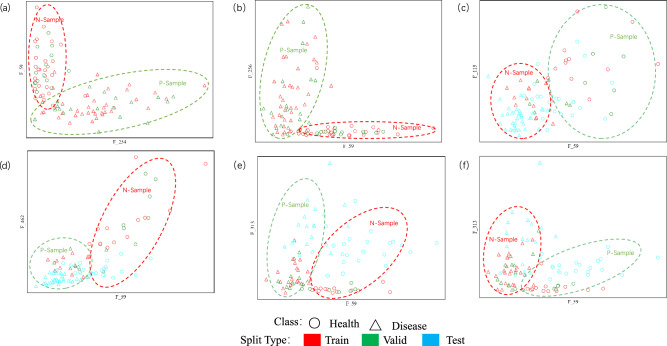
(**a**) Two batches of mixed samples on the Peak-area, the scatter plot visualization results of the optimal combination of the two features; (**b**) two batches of mixed samples on the Peak-height, the optimal combination of the two features Scatter plot visualization results; (**c**) 1Batch as the training set and validation set, 2Batch as the test set on the Peak-area, and the scatter plot visualization results of the optimal combination of the two features; (**d**) 1Batch as the training set and validation set, 2Batch is used as the test set on the Peak-height, and the optimal combination of the two features is used to visualize the results; (**e**) 2Batch is used as the training set and validation set, and 1Batch is used as the test set on the Peak-area, and the optimal combination of the two features is scattered. Dot plot visualization results; (**f**) 2Batch as the training and validation set; 1Batch as the test set on Peak-height, the optimal combination of the two features scatter plot visualization results.

From Fig. 4a,b, two batches of coronary heart disease mixed data sets, the characteristics corresponding to the two maximum chi-square values are drawn to draw a scatter plot. It can be seen that in the training set and the test set, the chi-square test method screened out the two. Each feature can distinguish coronary heart disease samples from healthy samples well. It is proved that the feature selection method used in this paper can deeply mine the significantly different biomarkers in the coronary heart disease data set and distinguish the two types of samples substantially. At the same time, the high-dimensional mass spectrometry data can find a combination of significantly different features, which can reduce the model's time complexity and improve the model's classification effect.

From Fig. 4c–f coronary heart disease batch mixed data set, the characteristics corresponding to the two most significant chi-square values are drawn into a scatter plot. It can be seen that on the Peak-area and Peak-height, the chi-square test is used to filter. The two features are optimally combined on the scatter plot. The two batches of samples are divided into two clusters, which proves that there are significant batch differences between batches 1 and 2 due to differences in collection time, which also increases the difficulty with which the model learns batch-by-batch variability.

### Dataset external in dependent test validation

By visualizing the results in the previous summary, it can be found that there is a significant difference between different batches in the coronary heart disease dataset. To verify the influence of the differences between the two batches of samples on the experimental results, this part of the experiment uses different batches of samples to test each other to verify the classification effect of the model. The coronary heart disease data set contains two batches of samples. This part of the experiment considers the differences between the two batches of samples. Based on the optimal parameters of the previous model, one batch of samples is used as the training and verification sets. One batch of samples is left as the test set to verify the classification effect of the model further.

The results in Table 7 show that on Peak-area and Peak-height when the two data sets are tested against each other, the lowest classification accuracy in the independent test set is 0.836. When using 2-Batch as training and validation sets, the classification accuracy is 0.950 on the 1-Batch separate test set. It is further proved that the method proposed in this paper still achieves a good classification effect on batches with significant differences, indicating that it is superior.

**Table 7:** Mutual verification results of first and second batches.

Batch	Peak type	Feature number	Best model	Valid	Test
1	Area	5	LR	1.000	0.933
	Height	4	Bagging	0.929	0.836
2	Area	4	Bagging	0.950	0.950
	Height	3	Bagging	0.952	0.927

### The optimal feature combination corresponds to the original M/Z interval

The significantly different mass-to-charge ratio intervals found by the method proposed in this paper have biological significance. By measuring the proteome expression profile and phosphorylated proteome profile of coronary heart disease and normal samples, it is possible to discover the precise treatment of coronary heart disease—potential biomarkers. Taking the feature subset corresponding to the optimal classification result in the mixed dataset when the sliding window step size is one as an example, two different features are found on Peak-area and Peak-height, Peak-area and Peak-height. The intersection is the F_59 difference feature. Using the feature to construct the index can determine the original cytoplasmic ratio interval and the biomarker of coronary heart disease. Formula 3.1 calculates the original feature interval through the feature through the sliding window calculation method. The calculation formula is as follows:

9 $$Original\_mass={\mathrm{F}}_{\mathrm{index}}*{\mathrm{W}}_{step}+{\mathrm{W}}_{start}$$

Among them, ${\mathrm{F}}_{\mathrm{index}}$ is the feature index after feature construction, ${\mathrm{W}}_{step}$ is the step size of the sliding window, and ${\mathrm{W}}_{start}$ is the starting point of the sliding window. Table 8 shows the original M/Z interval corresponding to the optimal features. Due to the large sampling dimension of mass spectrometry samples, it is difficult to analyze the difference information of a single mass-to-nucleus ratio in detail. Only the mass interval of salient features can be roughly obtained, and more detailed information cannot be obtained.

**Table 8:** The characteristics of the optimal classification result correspond to the original interval.

Peak type	Feature index	Chi-square value	Original mass
Area	254	33.262	308–309
	59	39.374	113–114
Height	256	32.267	310–311
	59	41.912	113–114

The original quality interval can be calculated by formula (9) by using the salient features F_59, F_254 and F_256 screened by the chi-square test of peak area and peak height in the revised paper. The smaller mass interval can provide ideas for the study of basic pathogenic mechanism, but the mass interval will contain many biological metabolite molecules, which can not better determine the significant pathogenic metabolite molecules. In order to better determine the significant pathogenic metabolite molecules, we searched for the significant pathogenic metabolite molecules by traversing the common mass values of all samples in the interval (in the process of mass analysis, substances with similar mass values belong to the same category, with three decimal places as the critical point here).

On the Peak-area and Peak-height, aiming at the remarkable feature F_59 corresponding to the original Mass interval 113–114, we traverse the common Mass values of all samples in the interval, and find that the mass value of 113.02 is the remarkable marker of healthy samples and sick samples, and the corresponding biological compounds can be obtained by combining metabolomics to compare the database in the later stage, thus revealing the pathogenesis of coronary heart disease.

### The effect of validating the model on another data

This part of the experiment uses the public colorectal liver metastases dataset to verify the classification results of the proposed method on other mass spectrometry datasets. In this part of the experiment, the training set and the test set are randomly divided according to the ratio of 7:3. By traversing the M/Z values in all colorectal liver metastases samples, the minimum and maximum M/Z values were 400 and 1600, which were used as the starting and ending values of the sliding window, respectively. Since the original data sparsity of the colorectal liver metastases is different from that of the coronary heart disease dataset, it is found that setting the sliding window step size parameter to 0.1 can effectively extract the effective original information of the colorectal liver dataset. Finally, the chi-square test is used for feature screening, model training is carried out on ten supervised learning models, and the classification effect is evaluated through various evaluation indicators.

The results in Table 9 show that in the peak area, when there are ten features, each evaluation index of the model is 1.000. At Peak-height, the classification accuracy is the highest at 0.944. It shows that the method proposed in this paper also applies to other mass spectrometry data and can achieve good classification results, which further verifies the feasibility of the proposed method.

**Table 9:** Classification results in the colorectal liver dataset.

Peak type	Best-model	Sn	Sp	Pre	Acc	MCC	F1	AUC
Area	RF/LR	1.000	1.000	1.000	1.000	1.000	1.000	1.000
Height	LR	0.889	1.000	1.000	0.944	0.894	0.941	0.975

## Conclusion

In this study, MSFS method is used to construct the features of the original mass spectrometry data, so as to realize the alignment operation of the original mass spectrometry data and reduce the redundant information and noise in the data. Chi-square test is used to select the least non-redundant feature combination, and the classification effect is verified on 10 models, and the feasibility of this method is verified on two public data sets. Different batches of sample mixtures were tested and verified internally on the coronary heart disease data set. When there are two features, the classification accuracy of peak area and peak height can reach 1.000, and the classification result is better than MetaboAnalyst software and the current experimental results. At the same time, chi-square test was used to find out two significantly different chi-square characteristics corresponding to two batches of samples in coronary heart disease data set, and visual analysis was made. It is found that the two batches of samples are quite different. External verification is carried out on different batches to verify the classification effect of the method proposed in this paper on samples with significant batch differences. The results show that the classification accuracy is as high as 0.950 when there are four features in the peak area, which verifies the method proposed in this paper. Feasibility. At the same time, the approximate interval of biomarkers of coronary heart disease can be further determined by mapping the two groups of features with the best classification results back to the original M/Z interval. Finally, the method proposed in this paper is verified on the data set of liver metastasis of colorectal cancer. When there are 20 features in the peak area, the classification accuracy can reach 1.000, which shows that the method proposed in this paper is feasible in mass spectrometry data sets (Supplementary Information S1).

### Supplementary Information

Supplementary Information.

## Data Availability

The datasets generated and/or analysed during the current study are available in the Proteome Xchange and Thermo Fisher Scientific repository. Dataset 1: https://datadryad.org/stash/dataset/doi:10.5061%2Fdryad.s8k81. Dataset 2: http://proteomecentral.proteomexchange.org/cgi/GetDataset?ID=PXD008383.
