# Supplementary material for: MSFC: a new feature construction method for accurate diagnosis of mass spectrometry data
Source: Sci Rep. 2023 Sep 21;13:15694. doi: 10.1038/s41598-023-42395-5 (PMC10514077; doi:10.1038/s41598-023-42395-5)
Supplement: Supplementary file 1 — Supplementary Information. [file 41598_2023_42395_MOESM1_ESM.zip › dataset/Supplementary material.docx]

Supplementary material

Due to the large size of the original data set, it is impossible to upload the original data set in official website. In order to facilitate reviewers to verify the authenticity of the data set, a link to the data set is provided.

CHD dataset: https://datadryad.org/stash/dataset/doi:10.5061%2Fdryad.s8k81.

CRLM dataset: http://proteomecentral.proteomexchange.org/cgi/GetDataset?ID=PXD008383.

The data set pretreated by MZmine2.53 is placed in the supplementary file to facilitate the verification of reviewers.
